# Microalgal Enzymes with Biotechnological Applications

**DOI:** 10.3390/md17080459

**Published:** 2019-08-05

**Authors:** Giorgio Maria Vingiani, Pasquale De Luca, Adrianna Ianora, Alan D.W. Dobson, Chiara Lauritano

**Affiliations:** 1 Marine Biotechnology Department, Stazione Zoologica Anton Dohrn, CAP80121 (NA) Villa Comunale, Italy; 2 Research Infrastructure for Marine Biological Resources Department, Stazione Zoologica Anton Dohrn, CAP80121 (NA) Villa Comunale, Italy; 3 School of Microbiology, University College Cork, College Road, T12 YN60 Cork, Ireland; 4 Environmental Research Institute, University College Cork, Lee Road, T23XE10 Cork, Ireland

**Keywords:** microalgae, enzymes, marine biotechnology, -omics technologies, heterologous expression, homologous expression

## Abstract

Enzymes are essential components of biological reactions and play important roles in the scaling and optimization of many industrial processes. Due to the growing commercial demand for new and more efficient enzymes to help further optimize these processes, many studies are now focusing their attention on more renewable and environmentally sustainable sources for the production of these enzymes. Microalgae are very promising from this perspective since they can be cultivated in photobioreactors, allowing the production of high biomass levels in a cost-efficient manner. This is reflected in the increased number of publications in this area, especially in the use of microalgae as a source of novel enzymes. In particular, various microalgal enzymes with different industrial applications (e.g., lipids and biofuel production, healthcare, and bioremediation) have been studied to date, and the modification of enzymatic sequences involved in lipid and carotenoid production has resulted in promising results. However, the entire biosynthetic pathways/systems leading to synthesis of potentially important bioactive compounds have in many cases yet to be fully characterized (e.g., for the synthesis of polyketides). Nonetheless, with recent advances in microalgal genomics and transcriptomic approaches, it is becoming easier to identify sequences encoding targeted enzymes, increasing the likelihood of the identification, heterologous expression, and characterization of these enzymes of interest. This review provides an overview of the state of the art in marine and freshwater microalgal enzymes with potential biotechnological applications and provides future perspectives for this field.

## 1. Introduction

Water covers around 71% of the Earth’s surface, with salt water responsible for 96.5% of this percentage [1]. Due to its molecular structure and chemical properties, water includes (and often participates in) every chemical reaction that is biologically relevant [2]. In such reactions, enzymes cover a fundamental role: They are organic macromolecules that catalyze biological reactions (so-called “biocatalysts” [3]). Due to their substrate-specificity, enzymes are commonly used in several sectors (such as food processing, detergent, pharmaceuticals, biofuel, and paper production) to improve, scale, and optimize industrial production. For example, hydrolases, which are enzymes that catalyze the hydrolysis of chemical bonds, have applications in several fields. Examples of industrially relevant hydrolases are cellulases for biofuel production [4], amylases for syrup production [5], papain, phytases and galactosidases for food processing [6], and other hydrolases which have various pharmaceutical applications [7]. The demand for new enzymes is growing every year, and many financial reports expect the global enzyme market value to surpass the $10 billion mark by 2024 (Allied Market Research, 2018, https://www.alliedmarketresearch.com/enzymes-market;ResearchandMarket.com, 2018, https://www.researchandmarkets.com/research/6zpvw9/industrial?w=4), of which $7 billion alone will be for industrial applications (BCC Research, 2018, https://www.bccresearch.com/market-research/biotechnology/global-markets-for-enzymes-in-industrial-applications.html).

**Figure 1:**
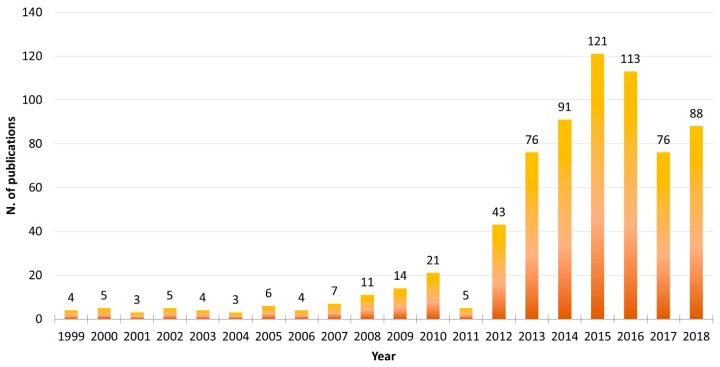
Microalgae Biotechnology PubMed Search Results 1999–2018. Using PubMed database search in the 20-years interval 1999–2018, the following search filters were set: The word “microalgae” in the [Title/Abstract] field and the word “biotechnolog*” in the [Text Word] field, using the asterisk (*) wildcard to expand the term selection (such as biotechnology, biotechnological, and biotechnologies).

Microalgae are photosynthetic unicellular organisms that can be massively cultivated under controlled conditions in photobioreactors with relatively small quantities of micro- and macro-nutrients [8], and can thus fit perfectly into this market sector. Microalgae continue to be used in a number of biotechnological applications. Searching the available literature in the PubMed database, this trend is clearly visible (search filters used were the word “microalgae” in the Title/Abstract field and the word “biotechnolog*” in the Text Word field, using the asterisk wildcard to expand the term selection; Figure 1). Considering the full 20-year interval between “1999–2018”, it is clear that as of 2012, there has been a rapid increase in the number of publications involving both “microalgae” and “biotechnology”, reaching a peak in the years 2015–2016.

The literature regarding the biotechnological applications of microalgae is dominated by four main research sectors: (1) Direct use of microalgal cells, for bioremediation applications and as food supplements [9]; (2) Extraction of bioactives for different applications (e.g., cosmeceutical, nutraceutical, and pharmaceutical applications, and for biofuel production [10,11]); (3) Use of microalgae as platforms for heterologous expression or endogenous gene editing and overexpression [12]; (4) Use of microalgae as sources of enzymes for industrial applications [13]. The latter field appears to be less well-studied compared to the others, due to the high costs currently involved in enzyme extraction and characterization, as well as the scarcity of annotated microalgal genomes.

Recent projects, such as those funded under the European Union Seventh Framework 2007–2017 (EU FP-7), e.g., BIOFAT (https://cordis.europa.eu/project/rcn/100477/factsheet/en) and GIAVAP (https://cordis.europa.eu/project/rcn/97420/factsheet/en), together with Horizon 2020 programs, e.g., ALGAE4A-B (http://www.algae4ab.eu/project.html) and VALUEMAG (https://www.valuemag.eu/), have resulted in an increase in –omics data (i.e., genomics, transcriptomics, proteomics and metabolomics data) available for microalgae, improving the possibility of finding new enzymes from both marine and freshwater species [14]. Mogharabi and Faramarzi recently reported the isolation of some enzymes from algae and highlighted their potential as cell factories [15]. This review aims to provide a summary of the current literature on microalgal enzymes with potential biotechnological applications with a particular focus on enzymes involved in the production of high-value added lipids and biodiesel, healthcare applications, and bioremediation.

## 2. Enzymes from Microalgae

### 2.1. Enzymes for High-Value Added Lipids and Biodiesel Production

**Figure 2:**
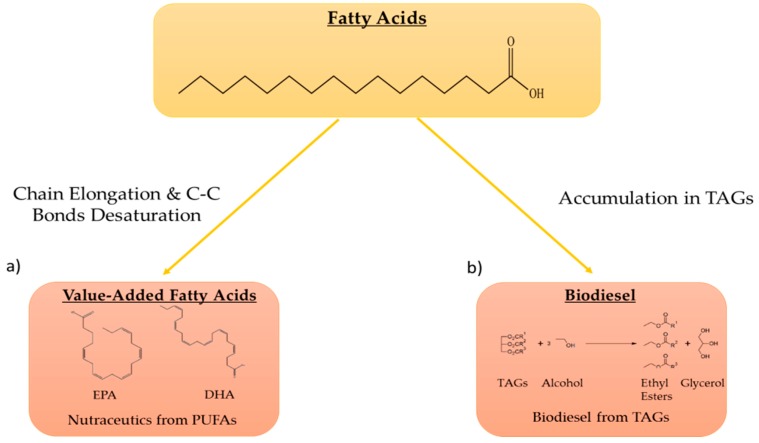
Examples of fatty acids of biotechnological interest. (**a**) Through various reactions of elongation and formation of double C-C bonds, poly-unsaturated fatty acids (PUFA) can be synthetized, such as eicosapentaenoic acid (EPA) and docosahexaenoic acid (DHA) with nutraceutical or food applications; (**b**) Accumulation in triglycerides (TAGs) and biodiesel formation via chemical transesterification.

Microalgae are known to accumulate large amounts of lipids [16], with triglycerides (TAGs) and poly-unsaturated fatty acids (PUFA) being the most studied from a biotechnological application standpoint, particularly for the production of biodiesel and nutraceuticals [9,16,17,18]. TAGs, esters derived from glycerol and three chained fatty acids (FA) which are usually stored in cytosol-located lipid droplets [19], can be used to produce biodiesel following acid- or base-catalyzed transesterification reactions [20]. PUFAs, for their part, have well-proven beneficial health effects [21,22], especially Ω-3 fatty acids such as docosahexaenoic acid (DHA) and eicosapentaenoic acid (EPA) (Figure 2).

The most frequently studied enzyme involved in lipid synthesis is acyl-CoA diacylglycerol acyltransferase (DGAT), involved in the final reaction of the TAG biosynthetic pathway [23,24]. Three independent groups of enzymes, referred to as acyl-CoA diacylglycerol acyltransferases type 1, 2, and 3 (DGATs 1-2-3), take part in the acyl-CoA-dependent formation of TAGs from its precursor sn-1,2-diacylglycerol (DAG) [25]. The individual contribution of each DGAT isoenzyme to the fatty acid profile of TAG differs between species [24,26].

A gene encoding DGAT1 was initially discovered in the green alga *Chlorella ellipsoidea* by Guo et al. [27], and an experiment involving overexpression of DGAT1 was subsequently performed in the oleaginous microalgae *Nannochloropsis oceanica* [28]. The first DGAT2 sequence was obtained from the green alga *Ostreococcus tauri* [29], and different studies involving overexpression of DGAT2 were performed. In particular, DGAT2 overexpression led to an increase in TAG production in the diatoms *Phaeodactylum tricornutum* [30] and *Thalassiosira pseudonana* [31], and in the oleaginous microalgae *Neochloris oleoabundans* [32] and *N. oceanica* [33]. Different isoforms of DGAT2 (NoDGAT2A, 2C, 2D) have successively been identified in *N. oceanica* and different combinations of either overexpression or under-expression have been analyzed. These combinations gave different fatty acid-production profiles, with some optimized for nutritional applications and others for biofuel purposes [34]. Even if the green alga *Chlamydomonas reinhardtii* is considered a common biofuel feedstock, it showed no clear trends following overexpression of different DGAT2 isoforms, with increased levels of TAG in some reports [35], while levels were not increased in others [36]. Recently, Cui and coworkers [37] characterized a dual-function wax ester synthase (WS)/DGAT enzyme in *P. tricornutum,* whose overexpression led to an accumulation of both TAGs and wax esters. This was the first report of this particular enzyme in a microalga, and a patent involving the enzyme was subsequently filed (Patent Code: CN107299090A, 2017). 

In addition to DGAT, other genes have been targeted in order to increase high-value added lipid production, including glucose-6-phosphate dehydrogenase (G6PD), ∆6-desaturase, 6-phosphogluconate dehydrogenase (6PGD), glycerol-3-phosphate acyltransferase (GPAT1-GPAT2), and acetyl-CoA synthetase 2 (ACS2). Overexpression of these enzymes resulted in increased lipid contents [38,39,40,41,42]. In particular, two patents for desaturases have been filed. One covers a Δ6-desaturase from *Nannochloropsis* spp., which converts linoleic acid to γ-linolenic acid (GLA) and α-linolenic acid (ALA) to stearidnoic acid (Patent Code: CN101289659A, 2010). The other covers a Ω6-desaturase from *Arctic chlamydomonas* sp. *ArF0006*, which converts oleic acid to linoleic acid (Patent Code: KR101829048B1, 2018).

**Figure 3:**
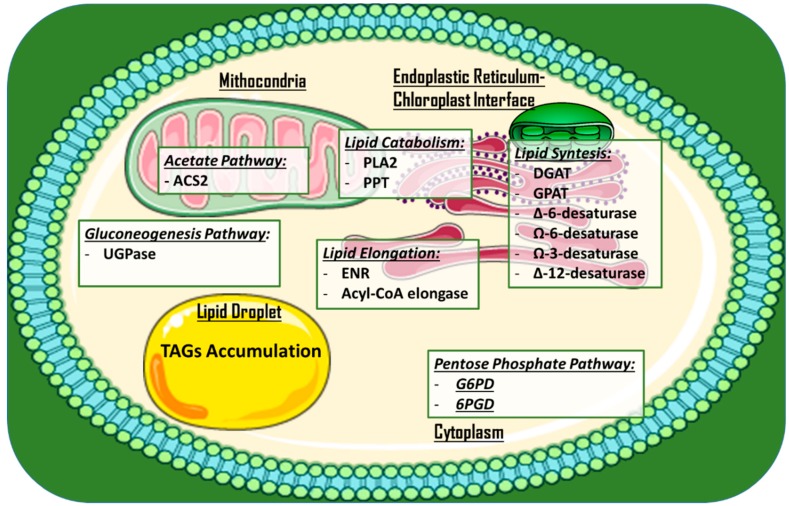
Main studied and engineered enzymes for TAGs and PUFAs in microalgae for the production of high value-added lipids. Enzymes are roughly divided in subcellular compartments. A single lipid droplet where TAGs are accumulated is added. Abbreviations: DGAT: Acyl-CoA diacylglycerol acyltransferase; G6PD: Glucose-6-phosphate dehydrogenase; 6PGD: 6-phosphogluconate dehydrogenase; GPAT: Glycerol-3-phosphate acyltransferase; ACS2: acetyl-CoA synthetase 2; PLA2: Phospholipase A2; ∆-6/∆-12-Desaturase: delta-6/delta-12 fatty acid desaturase; Ω-3/Ω-6-desaturase: omega-2/omega-6 fatty acid desaturase; ENR: Enoyl-acyl carrier protein reductase; UGPase: UDP-glucose pyrophosphorylase; TAG: Triglyceride.

Other approaches to increase lipid production and/or alter lipid profiles via gene disruption have been employed. Examples include the knock-out of a phospholipase A2 (PLA2) gene via CRISPR/Cas9 ribonucleoproteins in *C. reinhardtii* [43], microRNA silencing of the stearoyl-ACP desaturase (that forms oleic acid via addition of a double-bond in a lipid chain [44]) in *C. reinhardtii* [45], and meganuclease and TALE nuclease genome modification in *P. tricornutum* [46]. This last approach involved modifying the expression of seven genes, potentially affecting the lipid content (UDP-glucose pyrophosphorylase, glycerol-3-phosphate dehydrogenase, and enoyl-ACP reductase), the acyl chain length (long chain acyl-CoA elongase and a putative palmitoyl-protein thioesterase), and the degree of fatty acid saturation (Ω-3 fatty acid desaturase and ∆-12-fatty acid desaturase). In particular, a mutant for UDP-glucose pyrophosphorylase showed a 45-fold increase in TAG accumulation under nitrogen starvation conditions. Figure 3 provides an overview of the subcellular localization of metabolic pathways and engineered enzymes in the aforementioned examples.

Finally, Sorigué and coworkers [47] reported, for the first time, the presence of a photoenzyme named fatty acid photodecarboxylase (FAP) in *Chlorella variabilis* str microalgae. NC64A. FAP converts fatty acids to hydrocarbons and may be useful in light-driven production of hydrocarbons. It is worth mentioning that Misra et al. [48] have developed a database to catalogue the enzymes which have been identified as being responsible for lipid synthesis from available microalgal genomes (e.g., *C. reinhardtii*, *P. tricornutum*, *Volvox carteri*), called dEMBF (website: http://bbprof.immt.res.in/embf/). To date, the database has collected 316 entries from 16 organisms, while providing different browsing options (Search by: “Enzyme Classification”, “Organism”, and “Enzyme Class”) and different web-based tools (NCBI’s Blast software integrated, sequence comparison, Motif prediction via the MEME software). The enzymes discussed in this section are reported in Table 1.

### 2.2. Enzymes for Healthcare Application

**Figure 4:**
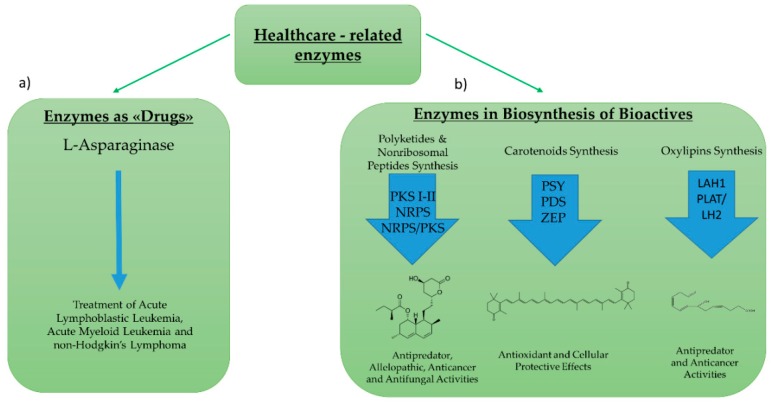
Enzymes for Healthcare Applications. Enzymes for healthcare applications can include: (**a**) Enzymes used directly as “drugs”, such as the l-asparaginase (**b**) enzymes involved in the biosynthetic pathway of active compounds, such as polyketides, carotenoids, or oxylipins. In the synthesis of polyketides, the enzymes studied are polyketide synthases and nonribosomal peptide synthases. For the synthesis of carotenoids, the most studied enzymes are phytoene synthase (PSY), phytoene decarboxylase (PDS) and zeaxanthin epoxidase (ZEP). For the synthesis of oxylipins the studied enzymes are lipoic acid hydrolases (LAH) and PLAT (Polycystin-1, Lipoxygenase, Alpha-Toxin)/LH2 (Lipoxygenase homology). An example of molecules and their roles for each pathway is also outlined.

Enzymes for healthcare applications can include: (1) Enzymes used directly as “drugs”, or (2) enzymes involved in the biosynthetic pathway of bioactive compounds (Figure 4). Regarding the first group, the most studied enzyme is l-asparaginase. l-asparaginase is an l-asparagine amidohydrolase enzyme used for the treatment of acute lymphoblastic leukemia, acute myeloid leukemia, and non-Hodgkin’s lymphoma [49]. Its hydrolytic effect reduces asparagine availability for cancer cells that are unable to synthesize l-asparaginase autonomously [50] l-asparaginase was historically first discovered and then produced in bacteria (e.g., *Escherichia coli*, *Erwinia aroideae*, *Bacillus cereus*) [51,52,53]. However, in order to overcome some of the economical and safety limits associated with marketing the enzyme [54,55], increased efforts began to focus on the identification and characterization of the enzyme in microalgae strains.

Paul [56] first purified an l-asparaginase in *Chlamidomonas* spp. with limited anticancer activity, and tested it in an in vivo anti-lymphoma assay. Ebrahiminezhad and coworkers screened 40 microalgal isolates via activity assays and reported on *Chlorella vulgaris* as a novel potential feedstock for l-asparaginase production [57]. 

Regarding enzymatic pathways involved in the synthesis of bioactive compounds, many studies have focused on polyketide synthases (PKS) and nonribosomal peptide synthetases (NRPS). PKS produce polyketides, while NRPS produce nonribosomal peptides. Both classes of secondary metabolites are formed by sequential reactions operated by these “megasynthase” enzymes [58,59]. Polyketides and nonribosomal peptides have been reported to have antipredator, allelopathic, anticancer, and antifungal activities [58,60,61,62]. PKS can be multi-domain enzymes (Type I PKS), large enzyme complexes (Type II), or homodimeric complexes (Type III) [63]. Genes potentially encoding these first two types’ of PKSs have been identified in several microalgae (e.g., *Amphidinium carterae*, *Azadinium spinosum*, *Gambierdiscus* spp., *Karenia brevis* [64,65,66,67]). Similarly, NRPSs have a modular organization similar to type I PKSs, and genes potentially encoding NRPSs have been found in different microalgae [68]. Moreover, metabolites that are likely to derive from hybrid NRPS/PKS gene clusters have been reported from *Karenia brevis* [69]. However, to our knowledge, there are no studies reporting the direct correlation of a PKS or NRPS gene from a microalga with the production of a bioactive compound.

Other microalgal enzymes which have been widely studied are those involved in the synthesis of compounds with nutraceutical and cosmeceutical applications, such as those involved in carotenoid synthesis (e.g., astaxanthin, β-carotene, lutein, and canthaxanthin). Carotenoids are isoprenoid pigments, which have many cellular protective effects, such as antioxidant effects occurring via the chemical quenching of O_2_ and other reactive oxygen species [70,71,72]. Their antioxidant properties can potentially protect humans from a compromised immune response, premature aging, arthritis, cardiovascular diseases, and/or certain cancers [72]. Among microalgae, the most studied for the industrial production of carotenoids are the halophile microalga *Dunaliella salina* and the green alga *Haematococcus pluvialis*, which naturally produce high amounts of carotenoids [73]. Moreover, *D. salina* is a particularly versatile feedstock, and many researchers have focused on obtaining maximum carotenoid yields without impeding its growth [74,75,76]. In addition, *D. salina* has been successfully transformed via different approaches, such as microparticle bombardment [77] or via *Agrobacterium tumefaciens* [78], increasing the feasibility of its use for biotechnological applications.

The most studied enzymes involved in carotenoid synthesis are: β-carotene oxygenase, lycopene-β-cyclase, phytoene synthase, phytoene desaturase, β-carotene hydroxylase, and zeaxanthin epoxidase [79]. In order to improve the production of carotenoids, different metabolic engineering approaches have been employed. The initial method used was to induce random or site directed mutations in an attempt to improve the activity of enzymes involved in the carotenoid metabolic pathway. Increased production of carotenoids can also be achieved by changing culturing conditions or by employing genetic modifications [79]. For example, mRNA levels of β-carotene oxygenase, involved in the biosynthesis of ketocarotenoids [80], increased in *Chlorella zofengiensis* under combined nitrogen starvation and high-light irradiation, and an increase canthaxanthin, zeaxanthin, and astaxanthin was observed [81]. Couso et al. [82] reported an upregulation in lycopene-β-cyclase, which converts lycopene to β-carotene [83] in *C. reinhardtii* under conditions of high light.

Regarding genetic modifications, Cordero [84] transformed the green microalga *C. reinhardtii* by overexpressing a phytoene synthase (which converts geranylgeranyl pyrophosphate to phytoene) isolated from *Chlorella zofingiensis*, resulting in a 2.0- and 2.2-fold increase in violaxanthin and lutein production, respectively. A phytoene desaturase, which transforms the colorless phytoene into the red-colored lycopene [85], was mutated in *H. pluvialis* by Steinbrenner and Sandmann [86], resulting in the upregulation of the enzyme and an increase in astaxanthin production. Galarza and colleagues expressed a nuclear phytoene desaturase in the plastidial genome of *H. pluvialis*, resulting in a 67% higher astaxanthin accumulation when the strain was grown under stressful conditions [87]. The insertion of a β-carotene hydroxylase from *C. reinhardtii* in *Dunaliella salina* resulted in a 3-fold increase of violaxanthin and a 2-fold increase of zeaxanthin [78]. The inhibition of *D. salina* phytoene desaturase using RNAi technology [88] resulted in an increase in phytoene content, but also a decrease in photosynthetic efficiency and growth rate.

More modern methods which have been used include the use of CRISPR/Cas9 (clustered regularly interspaced short palindromic repeats/CRISPR-associated protein 9) for precise and highly efficient “knock-out” of key genes [89]. For example, Baek et al. have used CRISPR/Cas9 to knock-out the zeaxanthin epoxidase (ZEP) gene in *C. reinhardtii* [90]. This enzyme is involved in the conversion of zeaxantin to violaxantin [91], and with its knock-out they obtained a 47-fold increase in zeaxanthin productivity. The current state-of-art involved in metabolic engineering for carotenoid production in microalgae is further discussed in other reviews [72,92].

Other studies have focused on enzymes involved in the synthesis of oxylipins, which are secondary metabolites that have previously been shown to have antipredator and anticancer activities [93,94,95]. Although oxylipin chemistry and putative biosynthetic pathways have been extensively studied in both plants and microalgae [96,97,98], the related enzymes and genes have only recently been identified and characterized in microalgae. Adelfi and coworkers have studied genes involved in the biosynthesis of oxylipins in *Pseudo-nitzchia multistriata* and performed transcriptome analysis on these genes in *Pseudo-nitzchia arenysensis* [99]. In diatoms, they characterized, for the first time, two patatin-like lypolitic acid hydrolases (LAH1) involved in the release of the fatty acid precursors of oxylipins and tested their galactolipase activity in vitro. Transcriptomic analysis also revealed three of seven putative patatin genes (g9879, g2582, and g3354) in *N. oceanica* and demonstrated that they were u-regulated under nitrogen-starvation conditions [100]. Similarly, Lauritano and coworkers analyzed the transcriptome of the green alga *Tetraselmis suecica* and reported three PLAT (Polycystin-1, Lipoxygenase, Alpha-Toxin)/LH2 (Lipoxygenase homology) domain transcripts [68]. The group also performed in silico domain assessment and structure predictions. The enzymes discussed in this section are described in Table 2.

### 2.3. Enzymes for Bioremediation

Bioremediation is the use of microorganisms and their enzymes for the degradation and/or transformation of toxic pollutants into less dangerous metabolites/moieties. The potential, which microalgae possess to proliferate in environments that are rich in nutrients (e.g., eutrophic environments) and to biosequestrate heavy metal ions, makes them ideal candidate organisms for bioremediation strategies [101,102]. The optimal goal in this area is to combine bioremediation activities with the possibility of extracting lipids and other high-value added compounds from the biomass that is produced [103,104,105,106] in order to reduce overall costs and to recycle materials. In this section, the focus will be on enzymatic bioremediation, which is a novel approach involving the direct use of purified or partially purified enzymes from microorganisms, and in this case, from microalgae, in order to detoxify a specific toxicant/pollutant [107]. This method has recently started to demonstrate promising results through the use of bacterial enzymes [108,109]. Examples are the use of enzymes for the bioremediation of industrial waste and, in particular, the recent use of chromate reductases found in chromium resistant bacteria, known to detoxify the highly toxic chromium Cr(VI) to the less-toxic Cr(III) [110].

In microalgae, a recent study focused on Cr(VI) reduction involving *C. vulgaris* [111]. This activity was suggested to involve both a biological route, through the putative enzyme chromium reductase, and a nonbiological route: Using the scavenger molecule glutathione (GSH). With respect to chromium removal, several strains of microalgae have been reported to be capable of achieving Cr(IV) removal from water bodies, including *Scenedesmus* and *Chlorella* species [112,113,114]. In the aforementioned transcriptome study on the green algae *Tetraselmis suecica*, a transcript for a putative nitrilase was reported [68]. Given that nitrilases are enzymes that catalyze the hydrolysis of nitriles to carboxylic acids and ammonia [115] and that this enzyme has recently been used for cyanide bioremediation in wastewaters [116], this nitrilase in *T. suecica* may prove to be useful in the treatment of cyanide contaminated water bodies. 

**Figure 5:**
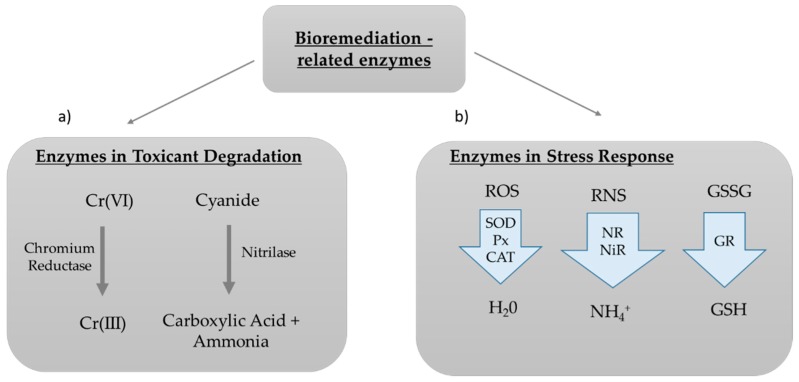
Enzymes for Bioremediation. Enzymes for Bioremediation can be: (**a**) Enzymes directly used for the degradation of toxicant compounds to less or non toxic versions (e.g., the hexavalent Chromium is converted to the less toxic trivalent Chromium due to the activity of Chromium Reductase); (**b**) Enzymes involved in cellular stress response mechanisms, such as peroxidases (Px), superoxide dismutase (SOD), and catalase (CAT) that detoxify reactive oxygen species (ROS), nitrate reductase (NR), and nitrite reductase (NiR) that detoxify reactive nitrogen species (RNS) in ammonium, and GR, that catalyzes the reduction of glutathione disulfide (GSSG) to glutathione (GSH).

Other enzymes have been reported to be overexpressed in microalgae when they are exposed to contaminants, but it is not clear whether or not they are directly involved in their degradation or whether they are produced as a stress defensive response in the cell in order to help balance cellular homeostasis (e.g., to detoxify reactive oxygen/nitrogen species produced after exposure to contaminants). Examples of these enzymes include peroxidases (Px), superoxide dismutase (SOD), catalase (CAT), and glutathione reductase (GR). SOD, Px, and CAT typically function in helping detoxify the cell from oxygen reactive species [117,118], while GR replenishes bioavailable glutathione, catalyzing the reduction of glutathione disulfide (GSSG) to the sulfhydryl form (GSH) [119]. Regarding the detoxification of reactive nitrogen species, the most studied enzymes in microalgae are the nitrate and nitrite reductases. The first enzyme reduces nitrate (NO_3_^−^) to nitrite (NO_2_^−^), while the second subsequently reduces nitrite to ammonia (NH_4_^+^). NH_4_^+^ is then assimilated into amino acids via the glutamine synthetase/glutamine-2-oxoglutarate amino-transferase cycle [120] (Figure 5).

For example, peroxidase activity has been reported in extracts from the green alga *Selenastrum capricornutum* (now named *Raphidocelis subcapitata* [121]), which was highly sensitive to very small concentrations of copper (Cu) (0.1 mM), and the authors proposed that the enzyme could be employed as a sensitive bioindicator of copper contamination in fresh waters [122]. Levels of Px, SOD, CAT, and GR have been reported to be upregulated following Cu contamination in *P. tricornutum* and following lead (Pb) contamination in two lichenic microalgal strains from the *Trebouxia* genus (prov. names, TR1 and TR9) [123,124]. In Morelli’s work, an increase of 200% in CAT activity indicated its important role in Cu detoxification. In contrast, Alvarez and coworkers reported that Px, SOD, CAT, and GR activity was higher in TR1 than in TR9 under control conditions (with the exception of CAT), while prolonged exposure to Pb resulted in the enzymatic activities of the two microalgae changing to similar levels, reflecting the different physiological and anatomical adaptations of the two organisms. TR1 possesses a thinner cell wall, thereby requiring it to have a more efficient basal enzymatic defence system, while TR9 has a thicker cell wall and induces the expression of intracellular defense mechanisms when the contaminant concentrations are high and physical barriers are no longer effective. Further studies will be required to assess whether these TR1 enzymes are more efficient than enzymes from other microalgal sources and the potential applications that these enzymes may have. All of the enzymes discussed in this section are reported in Table 3.

## 3. Conclusions and Future Perspectives 

Among aquatic organisms that have recently received attention as potential sources of industrially relevant enzymes [125,126], microalgae, in particular, stand out as a new sustainable and ecofriendly source of biological products (e.g., lipids, carotenoids, oxylipins, and polyketides). This review summarized the available information on enzymes from microalgae with possible biotechnological applications, with a particular focus on value-added lipid production, together with healthcare and bioremediation applications.

The promise of microalgae as potential sources of novel enzymes of interest is reflected in the abundance of recent reports in the literature in this area. However, the biotechnological exploitation of their enzymes in comparison to other potential sources has only become more feasible quite recently, primarily due to the implementation of novel isolation and culturing procedures, together with an increase in the availability of -omics data. This data has facilitated the use of a broader array of approaches, such as site-specific mutagenesis, bioinformatics-based searches for genes of interest, and/or the use of genome editing tools (e.g., CRISPR/Cas9 and TILLING), resulting in promising results particularly with respect to high-performance lipid [46] and carotenoid [89] production in different microalgae.

The majority of studies to date have focused on enzymes involved in pathways for lipid synthesis in order to increase their total production or to direct cellular production to lipid classes with applications as nutraceuticals, cosmeceuticals, or as a feedstock for biodiesel production. For this reason, several recent studies have focused on the improvement of lipid production in oleaginous microalgae. In addition, algal biomass is often used for the extraction of both lipids and other value-added products, such as pigments and proteins, in order to maximize the production of useful products such as these at the lowest possible cost [127,128,129].

Future approaches to maximize the enzymatic potential of microalgae are likely to focus on three different approaches: (1) The use of ever-increasing amounts of available -omics data to optimize microalgal strains for the production of valuable products, through the overexpression of one or more enzymes through the use of genome editing tools; (2) identification and subsequent characterization of metabolic pathways involved in the production of specific bioactives (e.g., polyketides), many of which are still poorly characterized; (3) the search for genes with direct biotechnological applications (e.g., l-asparaginase, chromate reductase, nitrilase) in microalgal genomes and transcriptomes datasets. A common element in all three approaches is the potential use of next generation sequencing based approaches (NGS) [130], the price of which is declining rapidly [131].

The feasibility of employing any of the aforementioned three approaches will be directly influenced by progress in methods to decrease the costs of growth and genetic manipulation of microalgae. The ultimate aim would be to mimic what has happened in the area of bacterial enzymology, where robust pipelines for enzyme discovery have been established. If this could be achieved, then it is clear that microalgae are likely to meet our expectations as a promising source of novel enzymes with utility in a variety of different biotechnological applications.

## Figures and Tables

**Table 1:** Enzymes from Microalgae for Lipid and Biodiesel Production. Marine and freshwater ecological strain sources are abbreviated as M or F, respectively. Algal classes of *Bacillariophyceae*, *Chlorophyceae*, *Trebouxiophyceae*, *Eustigmatophyceae*, *Mamiellophyceae*, *Coscinodiscophyceae*, and *Cyanidiophyceae* are abbreviated as BA, CH, TR, EU, MA, CO, and CY, respectively.

Ref.	Enzymes	Microalgae	Strain Source	Microalgal Class	Main Results
[39]	∆6-Desaturase	*Phaeodactylum tricornutum*	M	BA	Neutral lipid production enhanced and increase of EPA content
[41]	acetyl-CoA synthetase	*Chlamydomonas reinhardtii*	F	CH	Increase in neutral lipid production
[27]	acyl-CoA diacylglycerol acyltransferase 1	*Chlorella ellipsoidea*	F	TR	Sequence identification and function of TAG accumultation characterized
[28]	acyl-CoA diacylglycerol acyltransferase 1A	*Nannochloropsis oceanica*	M	EU	Increase in TAGs production both in nitrogen-replete and -deplete conditions
[36]	acyl-CoA diacylglycerol acyltransferase 2	*Chlamydomonas reinhardtii*	F	CH	No TAGs overproduction
[35]	acyl-CoA diacylglycerol acyltransferase 2	*Chlamydomonas reinhardtii*	F	CH	Five DGAT2 homologous genes identification and the overexpression of CrDGAT2-1 and CrDGAT2-5 resulting in a significant increase in lipid production
[33]	acyl-CoA diacylglycerol acyltransferase 2	*Nannochloropsis oceanica*	M	EU	Increase in neutral lipid production
[32]	acyl-CoA diacylglycerol acyltransferase 2	*Neochloris oleoabundans*	F	CH	Change of lipid profile
[29]	acyl-CoA diacylglycerol acyltransferase 2	*Ostreococcus tauri*	M	MA	Gene identification and enzyme characterization in heterologous systems
[30]	acyl-CoA diacylglycerol acyltransferase 2	*Phaeodactylum tricornutum*	M	BA	Increase in neutral lipid production with enrichment EPA-PUFAs content
[31]	acyl-CoA diacylglycerol acyltransferase 2	*Thalassiosira pseudonana*	M	CO	Increase in TAGs production with focus on the intracellular enzyme localization
[34]	acyl-CoA diacylglycerol acyltransferase 2A, 2C, 2D	*Nannochloropsis oceanica*	M	EU	Differential DGAT2 isoforms expression in different engineered strains with individual specialized lipid profiles
[47]	fatty acid photodecarboxylase	*Chlorella variabilis*	F	TR	Enzyme identification and alkane synthase activity tested
[38]	glucose-6-phosphate dehydrogenase	*Phaeodactylum tricornutum*	M	BA	Modest increase in neutral lipid production with a lipid composition switch from polyunsaturated to monounsaturated
[42]	glucose-6-phosphate dehydrogenase; phosphogluconate dehydrogenase	*Fistulifera solaris*	M	BA	Slight increase in TAGs production
[40]	glycerol-3-phosphate acyltransferase 1, 2	*Cyanidioschyzon merolae*	F	CY	Significant increase in TAGs production
[43]	phospholipase A2	*Chlamydomonas reinhardtii*	F	CH	Increase in TAGs production
[45]	stearoyl-ACP desaturase	*Chlamydomonas reinhardtii*	F	CH	Production of TAGs enriched in stearic acid
[46]	UDP-glucose pyrophosphorylase, glycerol-3-phosphate dehydrogenase, enoyl-ACP reductase, long chain acyl-CoA elongase, putative palmitoyl-protein thioesterase, Ω-3 fatty acid desaturase and ∆-12-fatty acid desaturase	*Phaeodactylum tricornutum*	M	BA	Significant increase in TAGs production (45-fold increase for UDP-glucose pyrophosphorylase mutant)
[37]	wax esther synthase/acyl-CoA diacylglycerol acyltransferase	*Phaeodactylum tricornutum*	M	BA	Increase in neutral lipids and wax esters production
**Patent Code (Year)**	**Enzymes**	**Microalgae**	**Strain Source**	**Microalgal Class**	**Notes**
CN107299090A (2017)	wax esther synthase/acyl-CoA diacylglycerol acyltransferase	*Phaeodactylum tricornutum*	M	BA	Neutral lipids and wax esters production enhanced
CN101289659A (2010)	∆6-Desaturase	*Nannochloropsis* spp.	M	EU	The enzyme sequence was identified and the enzyme characterized in bacterial systems
KR101829048B1 (2018)	Ω6-Desaturase	*Arctic Chlamydomonas* sp. *ArF0006*	F	CH	The enzyme sequence was identified and the enzyme characterized in bacterial systems

**Table 2:** Enzymes from Microalgae for Healthcare Applications. Marine, freshwater, and soil strain sources are abbreviated as M, F, or S, respectively. Algal classes of *Chlorophyceae*, *Trebouxiophyceae*, *Bacillariophyceae*, *Dinophyceae*, and *Chlorodendrophyceae*, are abbreviated as CH, TR, BA, DY, and CR respectively.

Reference	Enzymes	Microalgae	Strain Source	Microalgal Class	Main Results
[78]	β-carotene hydroxylase	*Dunaliella salina*	M	CH	Increase in violaxanthin and zeaxanthin production
[81]	β-carotene oxygenase	*Chlorella zofingiensis*	S	TR	Increase in canthaxanthin, zeaxanthin and astaxanthin production under combined nitrogen starvation and high light stress
[56]	l-asparaginase	*Chlamidomonas* spp.	F	CH	Enzyme purified and tested
[57]	l-asparaginase	*Chlorella vulgaris*	F, S	TR	Screening of 40 microalgal isolates searching for new l-asparaginase sources
[82]	lycopene-β-cyclase	*Chlamidomonas reinhardtii*	F	CH	Increased gene expression under high light stress
[99]	lypolitic acid hydrolase 1	*Pseudo-nitzschia multistrata, Pseudo-nitzschia arenysensis*	M	BA	Enzyme finding, characterization and retrieval of homologous sequences in other diatoms
[69]	non-ribosomal peptide synthase	*Karenia brevis*	M	DY	Gene cluster identification and chloroplastic localization identification
[68]	polycystin-1, Lipoxygenase, Alpha-Toxin/lipoxygenase homology 2	*Tetraselmis suecica*	M	CR	Three putative enzyme sequences identification and in silico domain assessment and structure prediction
[88]	phytoene desaturase	*Dunaliella salina*	M	CH	Increase in phytoene production
[84]	phytoene synthase	*Chlamidomonas reinhardtii*	F	CH	Increase in violaxanthin (2.0 fold) and lutein (2.2-fold) production
[86]	phytoene desaturase	*Haematococcus pluvialis*	F	CH	Increase in astaxanthin production
[87]	phytoene desaturase	*Haematococcus pluvialis*	F	CH	Increase in astaxanthin production
[64]	polyketide synthase	*Amphidinium carterae*	M	DY	Identification of a transcript coding for type I PKS β-ketosynthase domain
[65]	polyketide synthase	*Azadinium spinosum*	M	DY	Identification of type I PKS domains using a combination of genomic and transcriptomic anayses
[66]	polyketide synthase	*Gamberdiscus polynesiensis, Gamberdiscus excentricus*	M	CH	Identification of transcripts coding for type I and type II PKS domains
[67]	polyketide synthase	*Karenia brevis*	M	DY	Identification of eight transcripts, six of which coding for type I PKS catalytic domains
[90]	zeaxanthin epoxidase	*Chlamydomonas reinhardtii*	F	CH	Increase in zeaxanthin production of 47-fold

**Table 3:** Enzymes from Microalgae with utility in Bioremediation applications Marine, freshwater, and lichenic strain sources are abbreviated as M, F, and L respectively. Algal classes of *Trebouxiophyceae*, *Chlorodendrophyceae*, *Chlorophyceae*, and *Bacillariophyceae* are abbreviated as TR, CR, CH, and BA, respectively.

Reference	Enzymes	Microalgae	Strain Source	Microalgal Class	Main Results
[111]	Putative Cr Reductase	*Chlorella vulgaris*	F	TR	Enzymatic Cr conversion (from Cr(VI) to Cr(III)) detected
[68]	Nitrilase	*Tetraselmis suecica*	M	CR	Putative enzyme sequence identification
[122]	Putative ascorbate peroxidase	*Selenastrum capricornutum*	F	CH	High sensitivity to Cu concentration activity
[123]	superoxide-dismutase, catalase, glutathione reductase	*Phaeodactylum tricornutum*	M	BA	Higher detected enzymatic activity after Cu accumulation
[124]	superoxide-dismutase, catalase, glutathione reductase, ascorbate peroxidase	*Trebouxia 1 (TR1), Trebouxia 9 (TR9)*	L	TR	Constitutive higher enzymatic activity detected in TR1, while exposed to Pb brings TR1 and TR9 enzymatic activities to comparable levels
